# Study on the effect of mesenchymal stem cells on neural injury, inflammation and copper content in Wilson disease

**DOI:** 10.3389/fncel.2025.1648155

**Published:** 2025-12-15

**Authors:** Xiang-xue Zhou, Hao-ling Qin, Dingbang Chen, Jian Liao, Yinjie Liu

**Affiliations:** 1 Department of Neurology, The First Affiliated Hospital, Sun Yat-sen University, Guangdong Provincial Key Laboratory of Diagnosis and Treatment of Major Neurological Diseases, National Key Clinical Department and Key Discipline of Neurology, Guangzhou, China; 2 Department of Imaging, The First Affiliated Hospital of Sun Yat-sen University, Guangzhou, China

**Keywords:** Wilson disease, bone marrow mesenchymal stem cells, extrapyramidal neural network, oxidative stress, magnetic sensitive imaging, copper metabolism

## Abstract

**Objective:**

To investigate the effects of bone marrow mesenchymal stem cells (BMSCs) on extrapyramidal neural network of Wilson disease (WD).

**Methods:**

27 6-month-old toxic milk mice (TX mice, WD animal model) and 15 C57 mice were selected. Corrected phase (CP) value on susceptibility weighted imaging (SWI), fractional anisotropy (FA) on diffusion tensor imaging (DTI) were performed. The volume of fiber connections was determined. BMSCs was transplanted though tail vein injection (1 × 10^6^, 0.5 mL). The myelin basic protein (MBP), amyloid precursor protein (*β*-APP), nitric oxide (NO), glutathione (GSH) and interleukin (IL-1β) were determined at 1, 2, 4 and 8 weeks after transplantation.

**Results:**

The CP value of TX mice increased at 4 (*p* = 0.029) and 8 weeks (*p* = 0.037) after transplantation. FA values (*p* = 0.026, 0.020, 0.037) and the volume of neural fibers (*p* = 0.016, 0.023, 0.018) increased at 2, 4 and 8 weeks after transplantation. The pathological indexes of demyelination (MBP) and axon injury (*β*-APP) improved after BMSCs transplantation. The brain copper content decreased at 4 and 8 weeks after transplantation (*p* = 0.024, 0.038). The indexes of oxidative stress (NO and GSH) and inflammation (IL-1β) of TX mice were improved after transplantation.

**Conclusion:**

BMSCs can ameliorate WD extrapyramidal neural network injury. The mechanism may be related to reducing copper deposition and alleviating oxidative stress and inflammatory response.

## 1 Introduction

Wilson disease (WD) is an autosomal recessive disorder of copper metabolism caused by mutations in the ATP7B gene. WD patients have abnormal deposits of copper in the brain and liver (Zhou et al., 2016a). The main manifestations are liver cirrhosis, neuropsychiatric symptoms, and corneal Kayser-Fleischer ring (pigment ring caused by copper deposition in the cornea). The neurological symptoms of WD patients are mainly extrapyramidal symptoms such as dystonia, slurred speech, ataxia, involuntary movement. The accumulation of copper in WD was mainly concentrated in the extrapyramidal nuclei including basal ganglia, brainstem and cerebellum (Lao and Le, 2024; Le Doux, 2024). The connecting fibers between these brain nuclei formed extrapyramidal neural network. We have confirmed that there is extrapyramidal neural network injury in the brain (Zhou et al., 2016b), and the neural network injury has an important impact on the severity of neurological symptoms using diffusion tensor imaging (DTI) and resting state functional MRI (rs-fMRI) in WD patients and WD animal model toxic milk mice (TX mice) (Ono et al., 1997). Pathological changes of WD extrapyramidal neural network include metal deposition, demyelination and axonal injury, followed by neuronal necrosis in the later stage (Zhou et al., 2019).

At present, the treatment of WD is mainly to reduce the copper deposition. But even after standard copper excretion treatment, WD patients still have obvious neural damage, and still have obvious neuropsychiatric symptoms. Current treatments prevent further harm but cannot reverse or repair existing damage. Transduction of the ATP7B gene using recombinant adeno-associated virus can increase the synthesis of ATP7B protein, especially in the liver, and reduce copper deposition in organs (Padula et al., 2022; Padula et al., 2023). However, a reduction in brain copper deposition cannot effectively alleviate neurological damage in WD. This limits the application of gene therapy in the treatment of WD neurological symptoms. There is no effective method to improve extrapyramidal network injury (Lao and Le, 2024; Le Doux, 2024). Stem cells may bring new opportunities for WD treatment (Chen et al., 2013; Li et al., 2023; Zhangdi et al., 2023). Previous studies in our research group have shown that bone marrow-derived mesenchymal stem cells (BMSCs) can migrate to the brain and cause a decrease in brain copper (Chen et al., 2013). However, it is still unclear whether BMSCs can repair extrapyramidal neural network injury, and whether it has an effect on pathogenic factors such as oxidative stress and inflammatory factors. The purpose of this research was to study the improvement of the connections of extrapyramidal fiber networks in TX mice after BMSCs transplantation by means of imaging methods. The pathological indexes were used to study the effects of BMSCs on brain pathological injury. Through the changes of brain inflammation indexes and oxidative stress indexes, the mechanism of BMSCs on the improvement of extrapyramidal network function was explored.

## 2 Materials and methods

### 2.1 Animals

27 TX mice aged 6 months (14 males, 13 females. 3 for pre-transplantation stem cell part, 12 for stem cell transplantation part, 12 for control group) and 15 healthy 6 months C57 mice (8 males, 7 females.) were collected (TX mice were donated by Professor Julian Mercer of the School of Life and Environmental Sciences in Deakin University, Australia). The missense mutation, ATP7B locus A4066G in TX mice causes methionine in the 8th transmembrane region of ATP7B protein to become valine, the activity of ATP7B protein (ATP7B protein is involved in the synthesis of ceruloplasmin, which excretes copper from liver into the bile ducts) is reduced, and copper is deposited in the liver, producing physiological properties similar to WD (Ono et al., 1997). TX mice show elevated copper levels in the liver and brain, as well as abnormal liver enzymes and other manifestations of liver injury. The identification of TX mice was achieved through ATP7B gene testing. TX mice and C57 mice were fed on a standard diet. The present study followed experimental procedures approved by the of Sun Yat-sen University Clinical Research and Laboratory Animal Ethics Committee and in adherence to the ARRIVE 2.0 guidelines.

### 2.2 Methods

**Figure 1:**
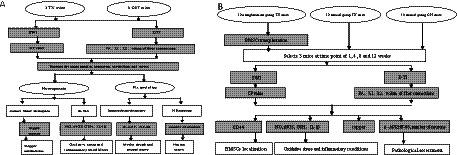
Experimental process diagram. **(A)** The experimental process before stem cell transplantation. **(B)** The experimental process after stem cell transplantation. DTI: diffusion tensor imaging; BMSCs: mesenchymal stem cells; SWI: susceptibility weighted imaging; CP: corrected phase; FA: fractional anisotropy; HE: hematoxylin–eosin staining; MBP: myelin basic protein; *β*-APP: amyloid precursor protein; NO: nitric oxide; iNOS: nitric oxide synthase; GSH: glutathione; IL-1β: interleukin.

The process of the experiment is shown in Figure 1.

#### 2.2.1 Imaging evaluation before transplantation

Susceptibility weighted imaging (SWI) was examined for TX mice and C57 mice. Four regions of interest (ROI) including basal ganglia, brain stem, cerebellum and cortex were selected. Outlined the ROI area by adding control points and manually checking the method. The setting of ROI was performed by two experienced neuroimaging physicians. The intensity and phase diagrams are obtained. The corrected phase (CP, a quantitative indicator of SWI imaging, to evaluate metal content) values were measured in each region of interest. 3.0 T Philips Achieva Nova Dual Plus superconducting MR Scanner and 8-channel SENSE Head coil was used. The scanning sequence is 3D-FFE sequence, TR/TE: 60 ms/40 ms, NSA: 1, FOV: 240x240mm2, FA: 18, resolution: 1mmx1mmx0.55 mm, echo shift: on. The original phase image is high-pass filtered by SPIN software, and the corrected phase map is obtained by dividing the original image by the filtered K-space data using a 64 × 64 low-pass filter.

DTI examination was performed on mice. The fractional anisotropy (FA, one of the quantitative indicators of DTI to evaluate nerve injury) values of each ROI and the eigenvalues λ1 and λ2 were measured, respectively. The regions with fibrous connections were located by DTI. The graph of fiber connections among brain nuclei was reconstructed and the volum of fiber connections was determined using method of fiber assignment continuous tracking (FACT). A single excited spin echo imaging sequence with 32 diffusion coding directions, TR 9500 ms, TE 70 ms, FOV 250 mm × 250 mm, matrix 128 × 128, layer thickness 2 mm, layer interval 0 mm, NSA = 1, was used. Diffusion weighting coefficients (B-values) were 0 and 1000s/mm^2^, respectively.

#### 2.2.2 Pathological evaluation before transplantation

Evaluation of neurons: TX mice and C57 mice were killed by intraperitoneal injection of 0.3% pentobarbital at a dose of 250 mg/kg. Brain tissue was removed in fresh after cardiac saline perfusion. The basal ganglia, brain stem, cerebellum and cortex were dissected. Half of the brain tissue was fixed with 4% paraformaldehyde, embedded in paraffin wax and sliced with a thickness of 5 μm. The number of neurons was measured by hematoxylin–eosin staining (HE) staining in fixed-size field of view. The Bioas Zool color pathological image analysis system was used to calculate the number of neuron body sections in 15,625 μm2 section at the same magnification (400 times objective lens).

Evaluation of myelin and axons: Half of the brain tissue was embedded with Optimal Cutting Temperature Compound (OCT) and frozen in liquid nitrogen and preserved. The brain tissue was sliced for protein determination. The myelin basic protein (MBP, indicators used for evaluating myelin injury) and amyloid precursor protein (*β*-APP, indicators used for evaluating axonal cable damage) was determined by immunohistochemical method. The slices were rinsed with PBS solution, sealed with normal goat serum, and incubated at 4 °C overnight after primary antibody was added. After rinsing with PBS, add secondary antibodies and incubate at 37 °C for about 1 h. The immunohistochemical primary antibody was rabbit anti-mouse monoclonal protein antibody (Guangzhou Seville Company, dilution 1:1000), and the secondary antibody was biotinylated sheep anti-rabbit IgG antibody (Guangzhou Seville Company, dilution 1:3000). The immunohistochemical results were analyzed by Imagepro-Plus 6.0 analysis and measurement software. The percentage of positive staining area and the average optical density (OD) were analyzed.

Evaluation of copper and biochemical indexes: Copper and biochemical indexes were determined after homogenization of brain nuclei. The brain copper was evaluated by flame method of atomic absorption spectrophotometer. The sample was introduced into the atomic absorption spectrophotometer, and the resonance line with a wavelength of 324.8 nm was absorbed after flame atomization. The absorption amount was proportional to the copper content and was quantified compared with the standard curve. The content of oxidative stress indicators nitric oxide (NO), nitric oxide synthase (iNOS), glutathione (GSH) and inflammatory response indicator interleukin (IL-1β) was detected using ELISA. The ELISA kits were provided by Guangzhou Seville Company (art. no. A013-2, A006-2, GEM0002).

#### 2.2.3 Stem cell cultivation and transplantation

15 mL femoral bone marrow fluid from mice (C57 mice aged 3 months in animal center) was collected and centrifuged. The supernatant was discarded and precipitated with complete medium. After the precipitate was centrifuged, the intermediate cell layer was absorbed, and PBS was added to clean the cells. The supernatant was discarded and the cell precipitation was retained. The cell suspension was inoculated into T25 bottles at a density of 1 × 10^6^/ml and cultured in a 5% CO2 incubator at 37 °C. After the fusion of the cells reached 80%, the cells were digested with pancreatic enzyme for 1–2 min and then cultured by subculture. The stem cells were cultured and expanded for two weeks to prepare for transplantation. Before transplantation, BMSCs identification was conducted: the cells grew adherently in a fibroblast-like manner. Trypan blue staining identified that the cell viability was good, and CD44 expression was positive.

BMSCs were injected into mouse tail vein under aseptic procedure. The total number of transplanted cells was 1 × 106, and the total injection suspension volume was 0.5 mL. The TX mice and C57 mice in the control group got a placebo (0.5 mL of normal saline) tail vein injection.

#### 2.2.4 Imaging and pathological evaluation after stem cell transplantation

Imaging evaluation: At each time point of 1, 2, 4 and 8 weeks after transplantation, 3 mice in the transplantation group and 3 mice in control group were selected for the following detection: DTI was performed, and FA, λ1 and λ2 values were determined. The fiber connections among brain nuclei were reconstructed and the number of fiber connections was determined. CP values on SWI were determined.

Stem cell localization evaluation: CD44 was detected by immunohistochemical method to evaluate the localization of stem cells (Zhou et al., 2016b) in brain tissue.

Pathological evaluation: The concentration of copper, MBP, *β*-APP, and the number of neurons in extrapyramidal brain nuclei were measured. The contents of IL-1β, NO, iNOS and GSH were measured.

#### 2.2.5 Comparison of TX mice and control group before and after transplantation

The imaging and pathological indexes of TX mice before and after transplantation, transplantation group and control group were compared to evaluate the effect of BMSCs transplantation. The inflammatory immune indexes and oxidative stress indexes were compared to explore the mechanism of stem cell transplantation.

### 2.3 Statistical analysis

Data are presented as mean ± standard deviation. The significance level was set at *α* = 0.05. T test was used to compare metal metabolism, pathological index and imaging index between TX mice and normal control mice. Imaging and pathological indexes before and after stem cell transplantation were compared by T test. Data with normal distribution were analyzed using Pearson analysis, while data with non-normal distribution were analyzed using Spearman analysis. SPSS21.0 software was used for statistical analysis.

## 3 Results

### 3.1 TX mice showed no neurological symptoms such as tremor and abnormal gait

TX mice were smaller than C57 mice of the same age, and their fur nutrition was poor. There was no significant improvement in body size and fur nutrition after stem cell transplantation.

### 3.2 Localization of BMSCs

**Figure 2:**
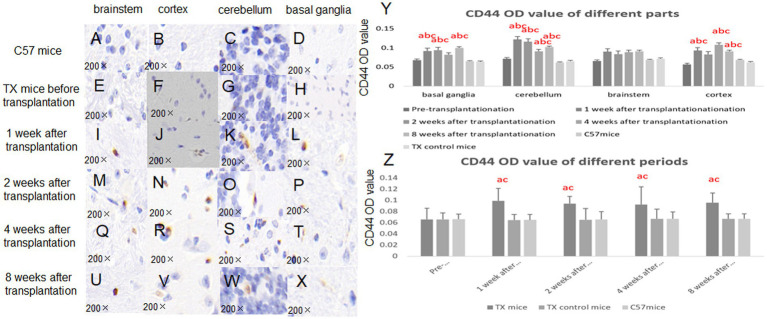
Immunohistochemical results of CD-44 in mouse brain before and after transplantation. CD-44 immunohistochemistry in C57 mice brain (**A**, brainstem; **B**, cortex; **C**, cerebellum; **D**, basal ganglia) did not show positive results. CD-44 immunohistochemistry in TX mice Brain (**E**, brain stem; **F**, cortex; **G**, cerebellum; **H**, basal ganglia) did not show positive results before transplantation. CD-44 immunohistochemistry in TX mice brain (**I**, brain stem; **J**, cortex; **K**, cerebellum; **L**, basal ganglia) showed brown stem cells 1 week after stem cell transplantation. CD-44 immunohistochemistry in TX mouse brain (**M**, brain stem; **N**, cortex; **O**, cerebellum; **P**, basal ganglia) showed brown stem cells 2 weeks after stem cell transplantation. CD-44 immunohistochemistry in TX mouse brain (**Q**, brain stem; **R**, cortex; **S**, cerebellum; **T**, basal ganglia) showed brown stem cells 4 weeks after stem cell transplantation. CD-44 immunohistochemistry in TX mouse brain (**U**, brain stem; **V**, cortex; **W**, cerebellum; **X**, basal ganglia) showed brown stem cells 8 weeks after stem cell transplantation; **Y**, CD44 optical density (OD) value in different parts of mice brain before and after stem cell transplantation; **Z**: CD44 OD value in different periods of mice brain before and after stem cell transplantation. The average OD reflects the content of the detected substance by detecting the absorbance value of the sample. *n* = 3 in each group. The results of the control TX mice group in **Y** are the average values at different time points. The results at each time point in **Z** are the average results of different parts. a: Compared with the normal control C57, the results are statistically significant (*p* ≤ 0.05); b: Compared with the TX mice before treatment, the results are statistically significant (*p* ≤ 0.05); c: Compared with the TX mice in the control group, the results are statistically significant (*p* ≤ 0.05). The statistical method adopted was Spearman analysis.

According to the immunohistochemical results of CD-44, CD-44 positive BMSCs in the brain of TX mice could be seen after injection of BMSCs (Figure 2). The OD value of CD-44 in TX mice was higher than that in C57 mice (*p* = 0.031, 0.019, 0.016, 0.020) and that in TX control mice (*p* = 0.041, 0.013, 0.026, 0.034) at 1, 2, 4, and 8 weeks after transplantation (Figure 2Z).

### 3.3 Imaging results of the connections of extrapyramidal networks before and after stem cell transplantation

**Figure 3:**
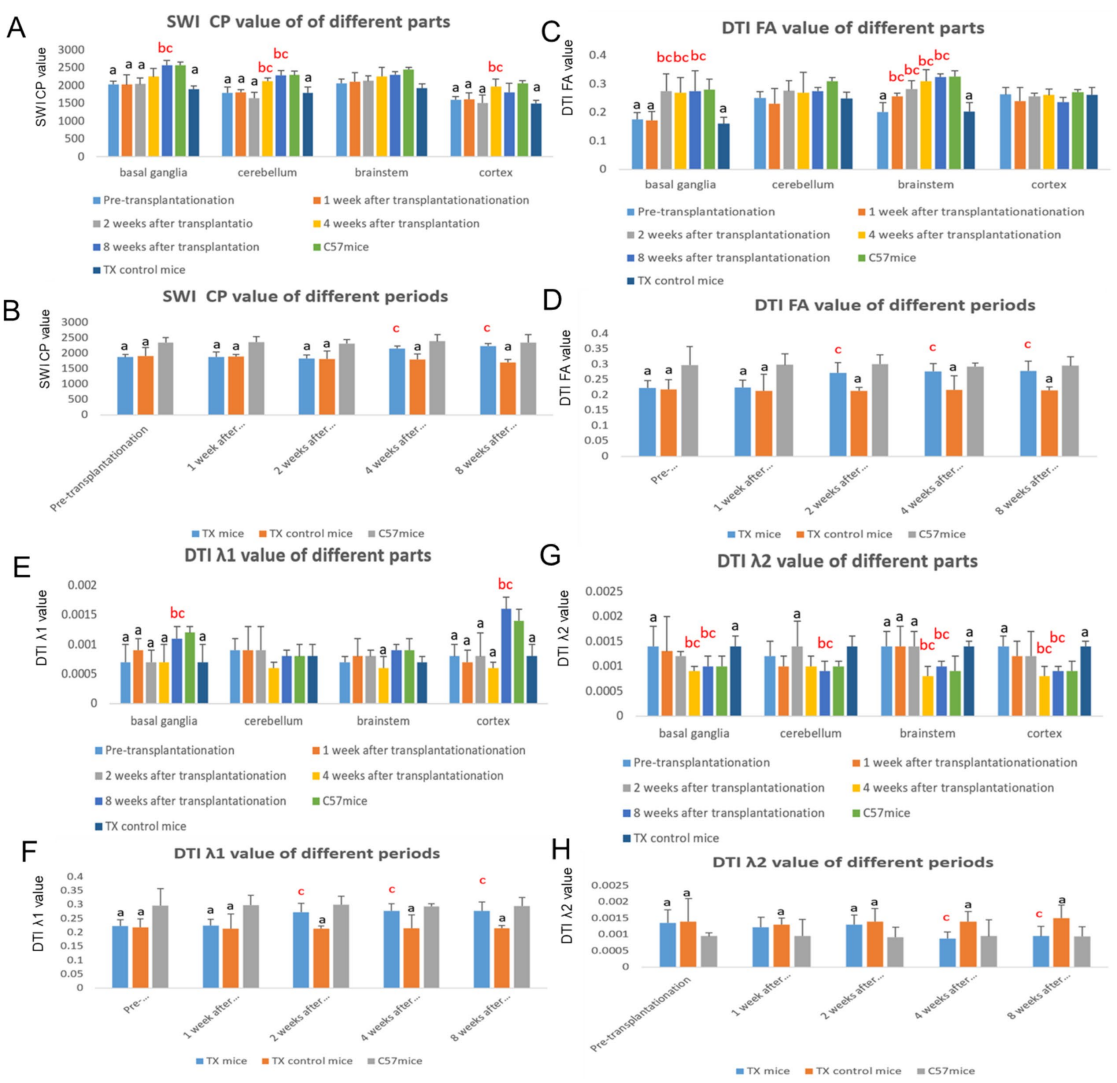
The changes of imaging values in mice brain before and after stem cell transplantation. **A** shows the change of SWI CP values in different parts of mice brain before and after stem cell transplantation. **B** shows the change of SWI CP values in different periods of mice brain before and after stem cell transplantation. **C** shows the change of DTI FA values in different parts of mice brain before and after stem cell transplantation. **D** shows the change of DTI FA values in different periods of mice brain before and after stem cell transplantation. **E** shows the change of DTI λ1 values in different parts of mice brain before and after stem cell transplantation. **F** shows the change of DTI λ1 values in different periods of mice brain before and after stem cell transplantation. **G** shows the change of DTI λ2 values in different parts of mice brain before and after stem cell transplantation. **H** shows the change of DTI λ2 values in different periods of mice brain before and after stem cell transplantation. *n* = 3 in each group. The results of the control TX mice group in **A, C, E, G** are the average values at different time points. The results at each time point in **B, D, F, H** are the average results of different parts. a: Compared with the normal control C57, the results are statistically significant (*p* ≤ 0.05); b: Compared with the TX mice before treatment, the results are statistically significant (*p* ≤ 0.05); c: Compared with the TX mice in the control group, the results are statistically significant (*p* ≤ 0.05). Statistical method for SWI CP values was Pearson analysis. The statistical method for DTI indexes was Spearman analysis.

CP values on SWI of TX mice before transplantation were lower than that in C57 mice. We detected upward trend of CP values of TX mice after transplantation. The CP values in TX mice was higher than that in TX control mice (*p* = 0.029, 0.037) at 4, and 8 weeks after transplantation, indicating the decrease of brain copper content (Figures 3A,B).

The FA values on DTI in TX mice before transplantation were lower than those of C57 mice. At 2, 4 and 8 weeks after transplantation (*p* = 0.026, 0.020, 0.037), the FA values of TX mice increased than that of TX control mice (Figures 3C,D). λ1 values in TX mice before transplantation were qualitatively lower than that of C57 mice. λ1 values of TX mice at 2, 4, 8 weeks after transplantation (*p* = 0.039, 0.030, 0.041) increased compared with those of TX control mice (Figures 3E,F). λ2 values in TX mice before transplantation were higher than those of C57 mice. λ2 values of TX mice decreased at 4 and 8 weeks after transplantation (*p* = 0.025, 0.027) than TX control mice (Figures 3G,H). These results suggest the improvement of nerve fiber axonal injury and demyelination.

**Figure 4:**
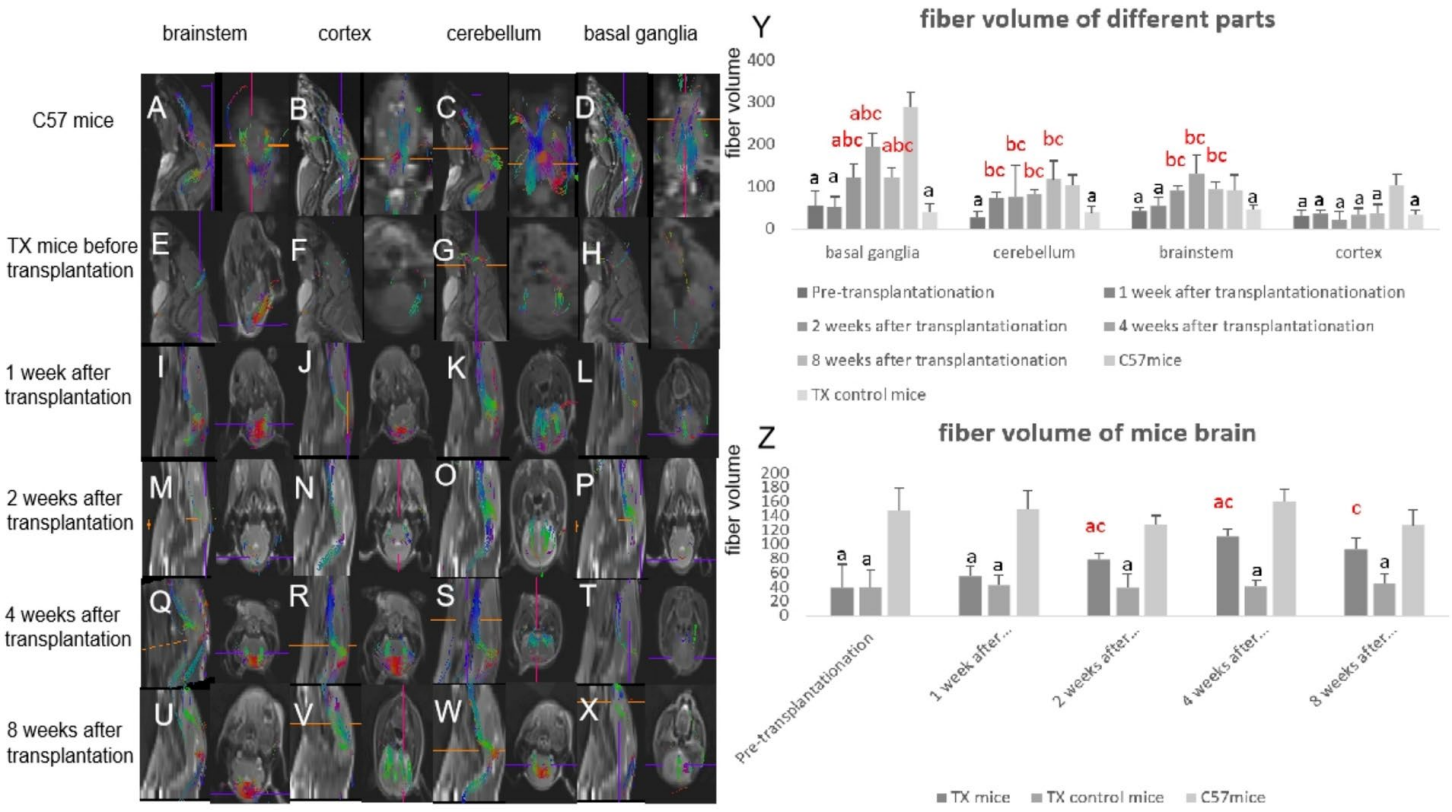
Imaging changes of neural fibers in mice brain before and after stem cell transplantation. The neural fibers in the brain of C57 mice are shown in **A** (cerebellum), **B** (brain stem), **C** (basal ganglia), **D** (cortex). The neural fibers of TX mice before stem cell transplantation are shown in **E** (cerebellum), **F** (brain stem), **G** (basal ganglia), **H** (cortex). The neural fibers of TX mice 1 week after stem cell transplantation are shown in **I** (cerebellum), **J** (brain stem), **K** (basal ganglia), **L** (cortex). The neural fibers of TX mice 2 weeks after stem cell transplantation are shown in **M** (cerebellum), **N** (brain stem), **O** (basal ganglia), **P** (cortex). The neural fibers of TX mice 4 weeks after stem cell transplantation are shown in **Q** (cerebellum), **R** (brain stem), **S** (basal ganglia), **T** (cortex). The neural fibers of TX mice at 8 weeks after stem cell transplantation are shown in **U** (cerebellum), **V** (brain stem), **W** (basal ganglia), **X** (cortex). The volume of neural fibers in mice brain before and after transplantation can be seen in **Y**. The volume of neural fibers in basal ganglia, cerebellum, brain stem after stem cell transplantation increased than that before transplantation. *n* = 3 in each group. **Y**, Neural fiber volume in different parts of mice brain before and after stem cell transplantation; **Z**: Neural fiber volume in different periods of mice brain before and after stem cell transplantation. The results of the control TX mice group in **Y** are the average values at different time points. The results at each time point in **Z** are the average results of different parts. a: Compared with the normal control C57, the results are statistically significant (*p* ≤ 0.05); b: Compared with the TX mice before treatment, the results are statistically significant (*p* ≤ 0.05); c: Compared with the TX mice in the control group, the results are statistically significant (*p* ≤ 0.05). The statistical method adopted was Spearman analysis.

The neural fiber volume in TX mice before transplantation was lower than that of C57 mice (Figure 4). The neural fiber volume of TX mice at 2, 4 and 8 weeks after stem cell transplantation (*p* = 0.016, 0.023, 0.018) increased than that of TX control mice (Figure 4). These results suggest the improvement of neural fiber damage after stem cell transplantation in TX mice.

### 3.4 Pathological and biochemical results before and after stem cell transplantation

**Figure 5:**
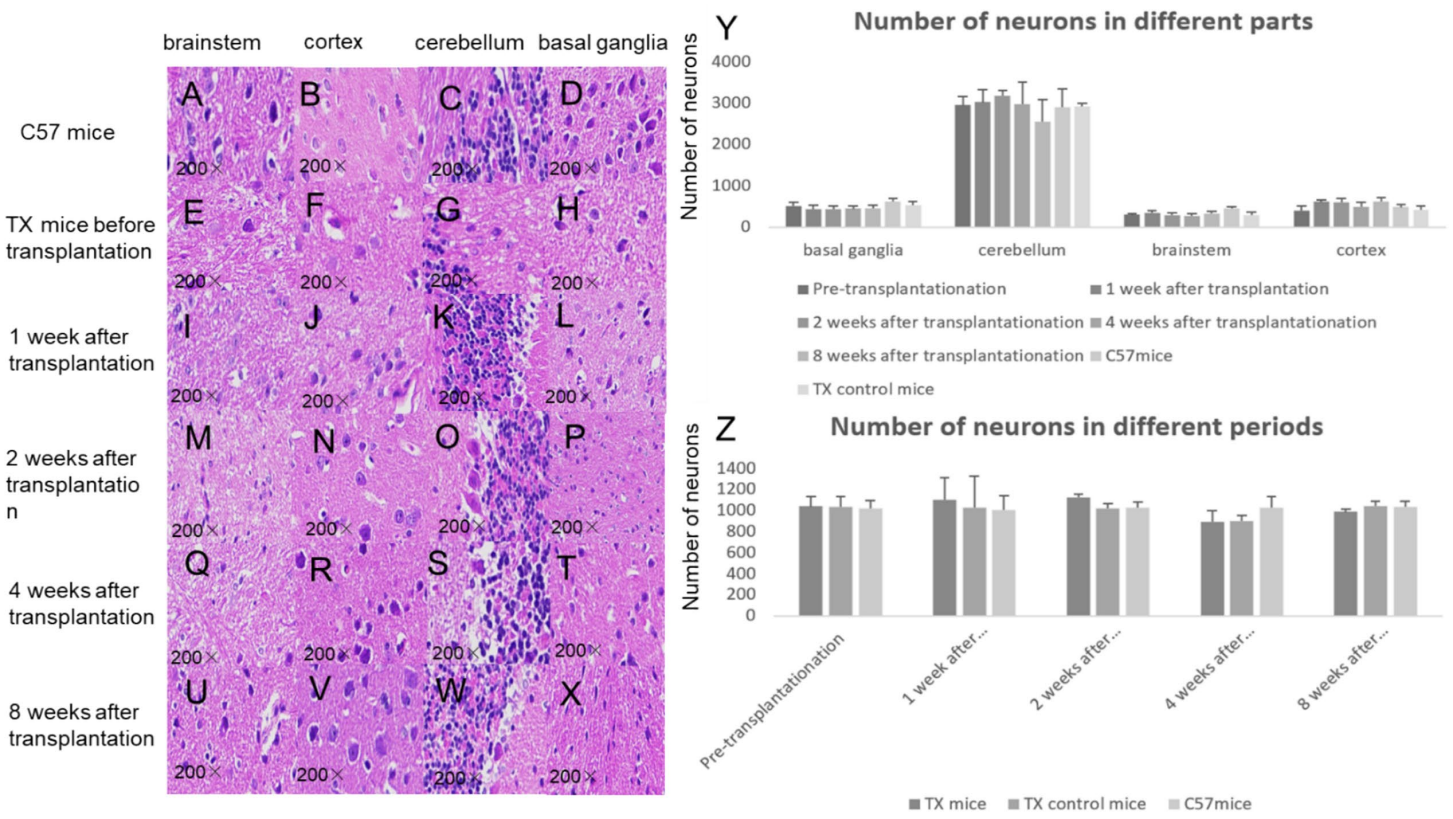
HE staining results of mice brain neurons before and after transplantation. HE staining of brain neurons of C57 mice is shown in **A** (brain stem), **B** (cortex), **C** (cerebellum), **D** (basal ganglia). HE staining of brain neurons of TX mice before stem cell transplantation is shown in **E** (brain stem), **F** (cortex), **G** (cerebellum), **H** (basal ganglia). HE staining of neurons 1 week after stem cell transplantation is shown in **I** (brain stem), **J** (cortex), **K** (cerebellum), **L** (basal ganglia). 2 weeks after stem cell transplantation, HE staining of neurons is shown in **M** (brain stem), **N** (cortex), **O** (cerebellum), **P** (basal ganglia). At 4 weeks after stem cell transplantation, HE staining of neurons is shown in **Q** (brain stem), **R** (cortex), **S** (cerebellum), **T** (basal ganglia). At 8 weeks after stem cell transplantation, HE staining of neurons is shown in **U** (brain stem), **V** (cortex), **W** (cerebellum), **X** (basal ganglia). **Y**, number of neurons in different parts of mice brain before and after stem cell transplantation; **Z**: number of neurons in different periods of mice brain before and after stem cell transplantation. *n* = 3 in each group. The results of the control TX mice group in **Y** are the average values at different time points. The results at each time point in **Z** are the average results of different parts. The statistical method adopted was Spearman analysis.

The number of neurons of TX mice after stem cell transplantation showed a mild tendency to grow than that before transplantation and TX control mice (Figure 5).

**Figure 6:**
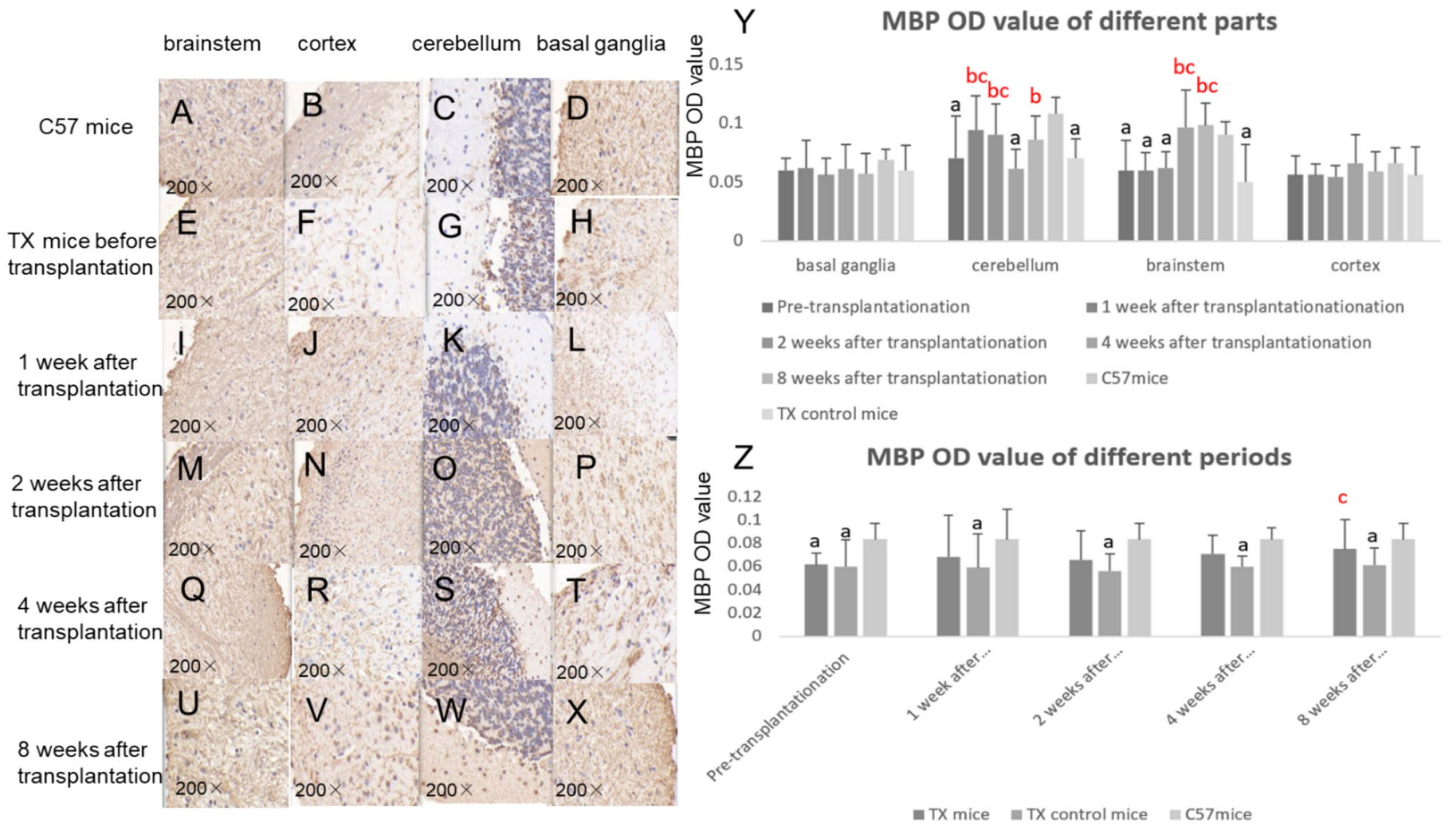
Myelin basic protein (MBP) immunohistochemical results of mice brain before and after transplantation. Immunohistochemistry of MBP in C57 mice brain is shown in **A** (brain stem), **B** (cortex), **C** (cerebellum), **D** (basal ganglia). The immunohistochemistry of MBP in TX mice before stem cell transplantation is shown in **E** (brain stem), **F** (cortex), **G** (cerebellum), **H** (basal ganglia). 1 week after stem cell transplantation, the MBP immunohistochemistry is shown in **I** (brain stem), **J** (cortex), **K** (cerebellum), **L** (basal ganglia). 2 weeks after stem cell transplantation, the MBP immunohistochemistry is shown in **M** (brain stem), **N** (cortex), **O** (cerebellum), **P** (basal ganglia). At 4 weeks after stem cell transplantation, the MBP immunohistochemistry is shown in **Q** (brain stem), **R** (cortex), **S** (cerebellum), **T** (basal ganglia). At 8 weeks after stem cell transplantation, the MBP immunohistochemistry is shown in **U** (brain stem), **V** (cortex), **W** (cerebellum), **X** (basal ganglia). **Y**, MBP OD value in different parts of mice brain before and after stem cell transplantation; **Z**: MBP OD value in different periods of mice brain before and after stem cell transplantation. *n* = 3 in each group. The results of the control TX mice group in **Y** are the average values at different time points. The results at each time point in **Z** are the average results of different parts. a: Compared with the normal control C57, the results are statistically significant (*p* ≤ 0.05); b: Compared with the TX mice before treatment, the results are statistically significant (*p* ≤ 0.05); c: Compared with the TX mice in the control group, the results are statistically significant (*p* ≤ 0.05). The statistical method adopted was Spearman analysis.

**Figure 7:**
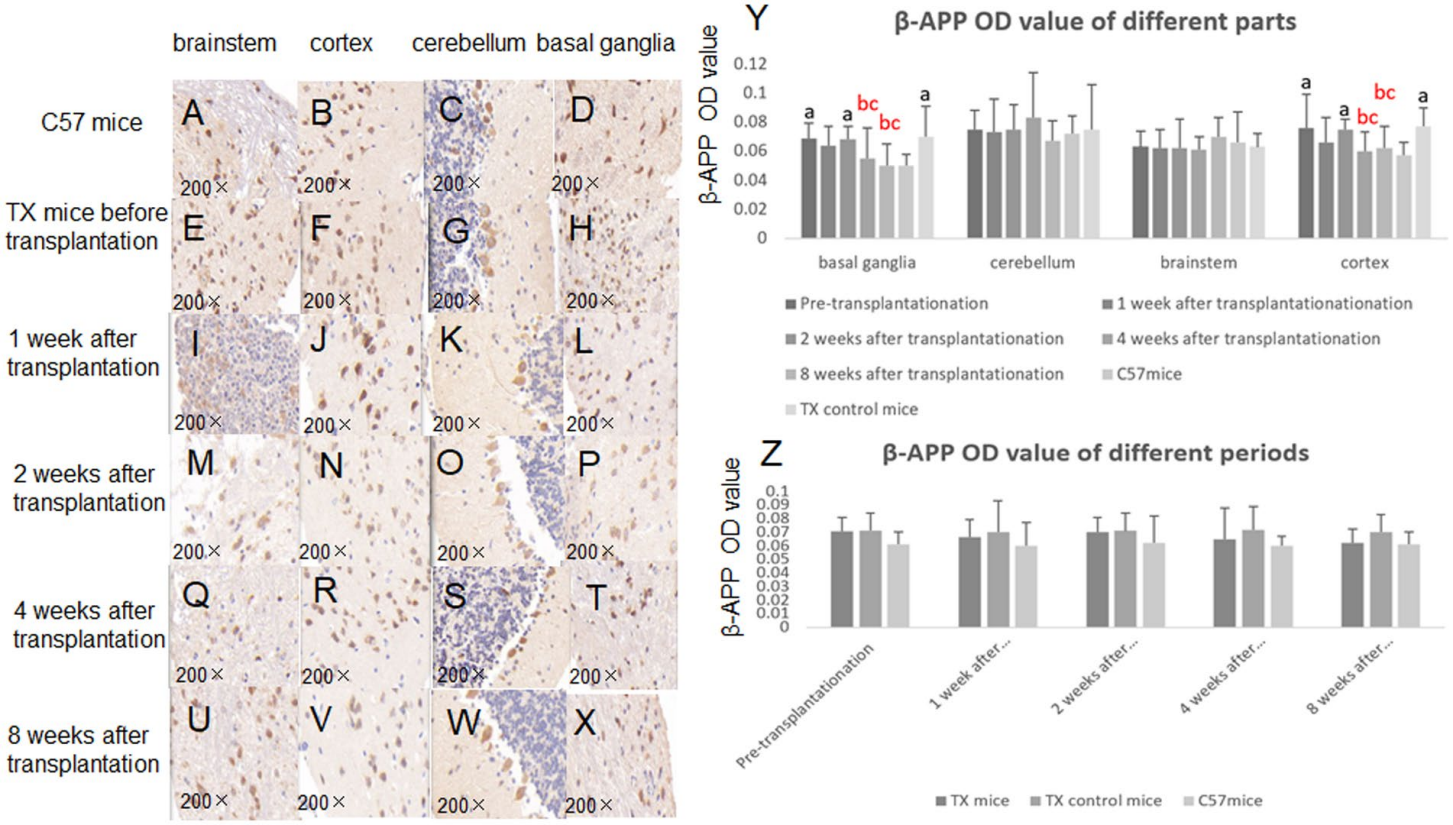
Immunohistochemical results of amyloid precursor protein (*β*-APP) before and after stem cell transplantation. Immunohistochemistry of β-APP in brain of C57 mice is shown in **A** (brain stem), **B** (cortex), **C** (cerebellum), **D** (basal ganglia). The immunohistochemistry of β-APP in brain of TX mice before stem cell transplantation is shown in **E** (brain stem), **F** (cortex), **G** (cerebellum), **H** (basal ganglia). 1 week after stem cell transplantation, the β-APPP immunohistochemistry is shown in **I** (brain stem), **J** (cortex), **K** (cerebellum), **L** (basal ganglia). 2 weeks after stem cell transplantation, the β-APP immunohistochemistry is shown in **M** (brain stem), **N** (cortex), **O** (cerebellum), **P** (basal ganglia). At 4 weeks after stem cell transplantation, β-APP immunohistochemistry is shown in **Q** (brain stem), **R** (cortex), **S** (cerebellum), **T** (basal ganglia). At 8 weeks after stem cell transplantation, the β-APP immunohistochemistry is shown in **U** (brain stem), **V** (cortex), **W** (cerebellum), **X** (basal ganglia). **Y**, β-APP OD value in different parts of mice brain before and after stem cell transplantation; **Z**: β-APP OD value in different periods of mice brain before and after stem cell transplantation. *n* = 3 in each group. The results of the control TX mice group in **Y** are the average values at different time points. The results at each time point in **Z** are the average results of different parts. a: Compared with the normal control C57, the results are statistically significant (*p* ≤ 0.05); b: Compared with the TX mice before treatment, the results are statistically significant (*p* ≤ 0.05); c: Compared with the TX mice in the control group, the results are statistically significant (*p* ≤ 0.05). The statistical method adopted was Spearman analysis.

The MBP OD values of TX mice before transplantation were lower than that of C57 mice. The MBP OD values at 8 weeks after transplantation (*p* = 0.033) increased compared with those of TX control mice, indicating the improvement of neural fiber demyelination (Figure 6). The *β*-APP OD values in TX mice before transplantation were qualitatively higher than that of C57 mice (Figure 7). β-APP OD values of basal segment (*p* = 0.037, 0.029) and cortex (*p* = 0.030, 0.036) at 4 and 8 weeks after transplantation decreased compared with those before transplantation (Figure 7), suggesting the reduction of axonal damage.

**Figure 8:**
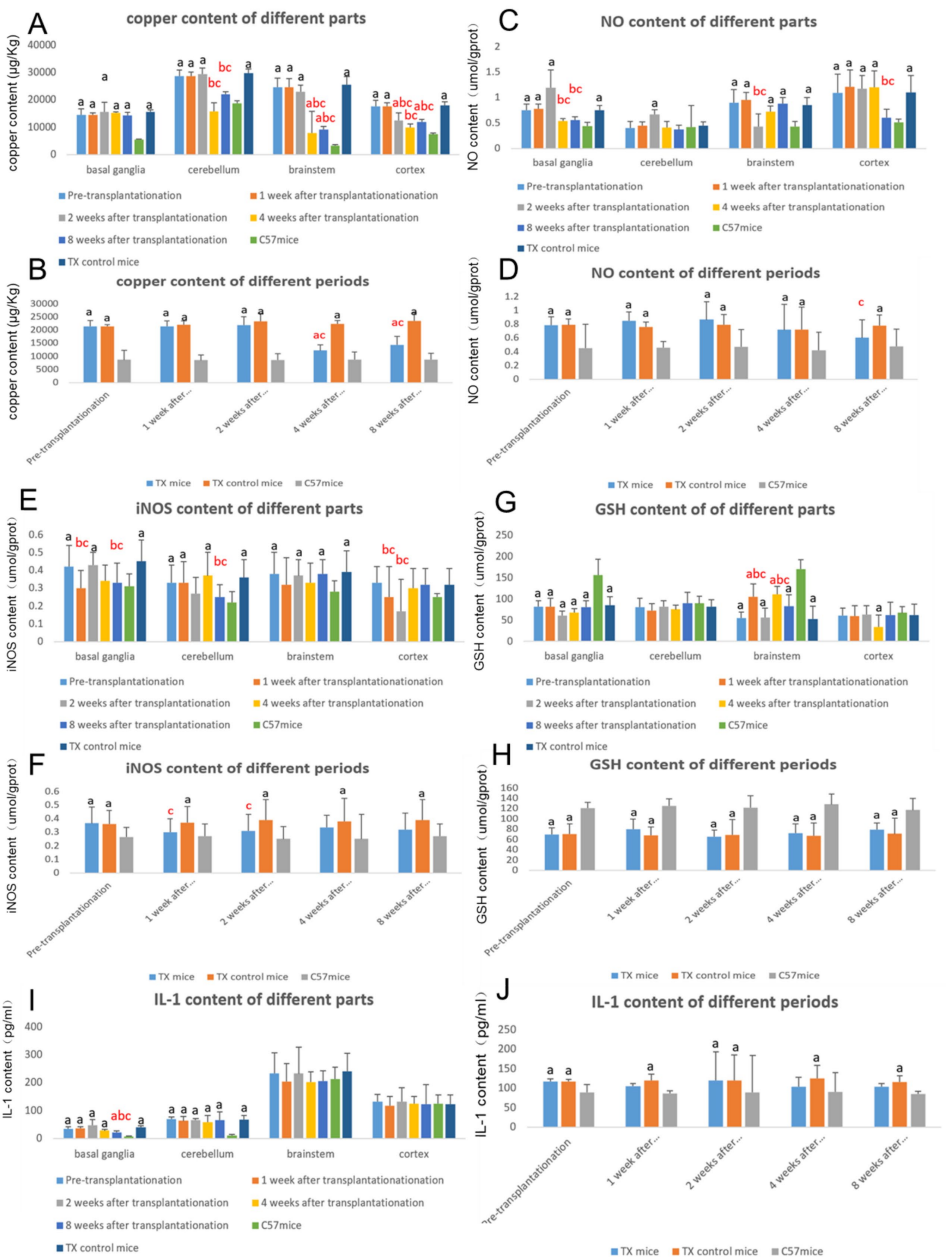
The changes of brain biochemical indexes before and after stem cell transplantation. **A** shows the copper content in different parts of mice brain before and after stem cell transplantation. **B** shows the copper content in different periods of mice brain before and after stem cell transplantation. **C** showed the NO content in different parts of mice brain before and after stem cell transplantation. **D** showed the NO content in different periods of mice brain before and after stem cell transplantation. **E** shows the iNOS content in different parts of mice brain before and after stem cell transplantation. **F** shows the iNOS content in different periods of mice brain before and after stem cell transplantation. **G** shows the GSH content in different parts of mice brain before and after stem cell transplantation. **H** shows the GSH content in different periods of mice brain before and after stem cell transplantation. **I** shows the IL-1 content in different parts of mice brain before and after stem cell transplantation. **J** shows the IL-1 content in different periods of mice brain before and after stem cell transplantation. *n* = 3 in each group. a: Compared with the normal control C57, the results are statistically significant (*p* ≤ 0.05); b: Compared with the TX mice before treatment, the results are statistically significant (*p* ≤ 0.05); c: Compared with the TX mice in the control group, the results are statistically significant (*p* ≤ 0.05). The statistical method for copper content was Pearson analysis. The statistical method for other indexes was Pearson analysis.

The brain copper content of TX mice was significantly higher than that of C57 mice. The copper content of TX mice at 4 and 8 weeks after transplantation (*p* = 0.024, 0.038) decreased compared with that of TX control mice (Figures 8A,B).

The NO content in TX mice before transplantation was qualitatively higher than that of C57 mice. The level of NO of TX mice at 8 weeks after transplantation (*p* = 0.037) decreased than that of TX control mice (Figures 8C,D). The iNOS content of TX mice of TX mice before transplantation was qualitatively higher than that of C57 mice. The iNOS content of TX mice at 1 and 2 weeks after transplantation (*p* = 0.015, 0.026) decreased than that of TX control mice (Figures 8E,F). The GSH content in TX mice before transplantation was qualitatively lower than that of C57 mice. The GSH content of brain stem in TX mice at 1 and 4 weeks after transplantation (*p* = 0.015, 0.026) increased than that of TX control mice (Figures 8G,H). These results suggest the reduction of oxidative stress and the increase of antioxidant capacity.

The level of IL-1β of TX mice before transplantation was qualitatively higher than that of C57 mice. The level of IL-1β after transplantation showed mild downward trend compared with that before transplantation and TX control mice (Figures 8I,J), indicating the reduction of inflammatory response.

## 4 Discussion

WD is a disorder of copper metabolism. Extrapyramidal neural network injury is the main cause of WD neurological symptoms (Zhou et al., 2016b). At present, the clinical treatment of WD is mainly to reduce the metal deposition in the brain. However, after copper expulsion treatment, the neurological symptoms of WD patients are still difficult to relieve. The extrapyramidal neural network damage is difficult to reverse, and the pathological changes such as demyelination and axonal injury are difficult to reduce. Stem cells may be an effective method for neural repair (Chen et al., 2013; Li et al., 2023; Zhangdi et al., 2023), but the therapeutic effect on extrapyramidal nerve injury in WD remains unclear. Our previous studies have shown that BMSCs can migrate to the brain and lead to a decrease in cerebral copper in TX mice (Chen et al., 2013). The purpose of this study was to study the effect of BMSCs on extrapyramidal neural network injury in WD. The effect of BMSCs on copper deposition and neural injury of TX mice was studied by pathological and immunohistochemical method. Through the changes of inflammatory factors and oxidative stress indexes, the mechanism of the improvement of WD neural injury by BMSCs was explored. TX mice is an ideal animal model for studying WD. In previous studies, we have confirmed that due to the expression defect of ATP7B, TX mice may show significant elevated liver copper and abnormal liver function in the early stage, and TX mice over 6 months old may show brain copper deposition and neuropathic injury (Zhou et al., 2019). However, TX mice do not have obvious symptoms in terms of motor function. Therefore, this study mainly investigated the improvement effect of BMSCs on neural injury in TX mice through imaging and pathological methods. Since obvious nerve damage occurred in 6-month-old TX mice (Zhou et al., 2019), we chose this time point to perform BMSCs transplantation and verify its repair effect on nerve damage. At each time point after transplantation, we set up placebo injection (without stem cell transplantation) TX mice and normal C57 mice as control groups to compare the state of BMSCs transplantation with the state of natural course of neural injury in TX mice and the state of neural aging in normal mice. The cell dose for BMSCs transplantation was based on our previous studies (Chen et al., 2013). The transplantation method adopted was tail vein injection. The reason is that injection into the lateral ventricle may interfere with the evaluation of neural injury.

We chose BMSCs for transplantation because BMSCs of the same species do not trigger strong immune response as other highly differentiated cells. We used CD44 immunohistochemistry to track and locate BMSCs (Liu et al., 2021). We found that CD44-positive cells could be found in the brain 1, 2, 4, and 8 weeks after stem cell injection, suggesting that BMSCs could migrate to the brain by injected through the tail vein. And BMSCs may be colonized for at least 8 weeks. Previous studies have found that stem cells may persist in the brain for a long period of time, and the presence of stem cells can be detected up to 120 days after transplantation (Amadeo et al., 2022). BMSCs that migrate into brain can differentiate into neurons in several weeks (Zhao et al., 2023). In this research, we did not confirm the integration of BMSCs through electrophysiological methods, nor use biological markers to prove the transformation of BMSCs into neurons. Therefore, it is not possible to confirm whether BMSCs promote the repair of nerve injury through paracrine action or directly replace the damaged nerve function through integration and transformation. The next step should detect biological markers of integration and transformation.

Through DTI and rs-fMRI studies, we confirmed the existence of extrapyramidal neural network injury in WD patients, and extrapyramidal network injury has important impact on the severity and symmetry of WD neurological symptoms (Zhou et al., 2016a; Zhou et al., 2016b). We observed that tremor was associated with the cerebello-thalamo-cortical network, and the inflow path of the globus pallidum was associated with hypokinesia including tremor, choreic movement and ataxia. Meanwhile the outflow path of the globus pallidum and substantia nigra may be an important cause of hypermyotonia (Zhou et al., 2016a; Zhou et al., 2016b). The treatment of extrapyramidal network injury is the key to alleviate WD neurological symptoms. Previous correlation analysis between DTI index and brain pathological index of TX mice confirmed that DTI FA value can reflect both axon and myelin injury, λ1 value can reflect the integrity of axon, λ2 and λ3 value can reflect the integrity of myelin (Zhou et al., 2019). FACT method is also an effective way to study fiber connections between brain nuclei. DTI is helpful in the identification of Parkinson’s disease, multi-system atrophy, and supranuclear atrophy (Cheng et al., 2023; Chen et al., 2023). Through the reconstruction of extrapyramidal fiber network, we found that the FA value and the volume of neural fiber in the brain of TX mice were lower than normal, which confirmed the injury of extrapyramidal neural network. After BMSCs transplantation, the FA value, λ1 value and the volume of neural fibers in the basal ganglia, brain stem, cerebellum and cortex of TX mice increased, λ2 value decreased, suggesting that BMSCs can repair the damage of WD extrapyramidal neural network.

Neurological symptoms of WD are not directly related to copper deposits in the brain. Secondary pathological damage caused by copper deposition may be the basis of neurological symptoms. Through previous pathological study of TX mice, we found that the pathological changes of WD brain were different in different disease stages. In the early stage of the disease, copper deposition is predominant. As the disease progresses, demyelination and axonal damage occurs. There is obvious neuronal death in the later stage (Zhou et al., 2016b; Zhou et al., 2014). Pathological damage can be evaluated by biological indicators. *β*-APP is a pathological marker of axonal injury (Sharma et al., 2023). MBP can reflect the myelin integrity of nerve fibers (Widder et al., 2020; Zhan et al., 2023). In this research, we found that the β-APP content increased and the MBP content decreased in TX mice, suggesting the axonal damage and demyelination in WD extrapyramidal system. After BMSCs transplantation, the content of β-APP showed downward trend, and the content of MBP in TX mice showed upward trend, indicating that the axonal injury and demyelination were improved. The number of neurons in TX mice showed no significant difference compared with C57 mice, which was related to the late occurrence of neuron death in WD mice. We observed a mild increase in the number of neurons after BMSCs transplantation, but the trend was not significant. It may not be obvious that stem cells promote neuron regeneration in the short term. At the same time, we cannot distinguish whether the increase in the number of neurons is the fusion of transplanted stem cells or the regeneration of neurons. Further research is needed.

SWI can be used as a non-invasive method to determine substance concentration (Ceballos-Ceballos et al., 2020). Our previous studies have confirmed that the CP value of SWI in WD is negatively correlated with the content of copper and iron (Zhou et al., 2019). Through SWI studies, we found that CP values in the substantia nigra, caudate nucleus, putamen, thalamus, cerebellum of WD patients were lower than normal, suggesting metal deposition in extrapyramidal nuclei (Zhou et al., 2014). In this research, CP values in the brain of TX mice were lower than that of C57 mice, also confirming the existence of copper deposition in WD brain. The increase of copper content in the brain was also confirmed by flame absorption metal determination in TX mice. The copper deposition in WD brain was selective, and the copper deposition in extrapyramidal system was more obvious. Multiple experiments have confirmed that BMSCs transplantation has significant effect on the improvement of copper content in WD liver and brain (Chen et al., 2013; Kerkar and Rana, 2022; Xiong et al., 2024; Hadrian et al., 2025). We confirmed that the CP value on SWI of TX mice after BMSCs transplantation increased, indicating a decrease of copper content. Flame atomic absorption assay also confirmed that the brain copper content decreased after transplantation. Our results suggest that BMSCs transplantation can promote the reduction of copper deposition in WD brain. The reason of the reduced copper deposition may be that stem cell leads to increased expression of ATP7B protein in the brain, which promotes copper excretion. The next step should be to investigate the mechanism of copper excretion after transplantation.

Copper deposition is not the only cause of neural damage in WD. In different stages of WD, there are other pathogenic factors. WD patients also have oxidative and inflammatory damage (Forman and Zhang, 2021; Chen et al., 2022; Aggarwal and Bhatt, 2020; Simon et al., 2020). We found that with the progression from metal deposition stage to fiber damage stage and neuron necrosis stage, the index of oxidative stress increased in WD patients abnormally. The degree of abnormal oxidative stress was consistent with the severity of neurological symptoms. The iNOS and NO contents may be sensitive indexes for evaluating oxidative stress of WD (Xiangxue et al., 2021). The GSH system is an important free radical scavenging system in the brain (Puig-Pijuan et al., 2022). The content of GSH decreased in WD patients with neuron necrosis stage. In addition to oxidative stress, WD patients also have inflammatory responses during the neuronal necrosis stage. Increased IL-1β levels can be detected (Simon et al., 2020). In this research, the levels of NO, iNOS and IL-1β in the brain of TX mice were higher than those of C57 mice, and GSH was lower, which confirmed the abnormal oxidative stress and inflammation in WD. After BMSCs transplantation, NO, NOS and IL-1β showed downward trend and GSH showed an upward trend in the brain of TX mice, suggesting that BMSCs can improve the abnormal oxidative stress and inflammation. Copper is a cofactor of Cu-Zn-superoxide dismutase (Cu-Zn-SOD). However, SOD was not used as index of oxidative stress in this study according to our previous studies in which NO, NOS and GSH were sensitive indicators while SOD decreased in WD, but did not reach statistical significance (Liu et al., 2021; Xiangxue et al., 2021). Our study suggests that the mechanism of BMSCs ameliorating extrapyramidal neural network injury in WD may be promoting copper elimination, alleviating oxidative stress and inflammatory response.

This paper suggests that BMSCs can cause imaging and pathological changes in the brain of TX mice. These changes are associated with a reduction in brain copper and a lessened oxidative stress and inflammatory response. The mechanism is still under our research. Based on our current results, the mechanism of BMSCs affecting copper metabolism and oxidative stress has the following reasons: 1. BMSCs can promote the expression of ATP7B and facilitate the excretion of copper in the brain. 2. After stem cell transplantation, the function of the glial lymphatic system improves, promoting the excretion of copper in the brain. 3. The increase in copper excretion alleviates oxidative stress in nerve cells and reduces copper death. These mechanisms still require further research data for confirmation.

There are the following problems in this study: 1. The observation time after BMSCs transplantation is not long. We observed BMSCs at 1, 2, 4, and 8 weeks after transplantation. A longer period of time should be observed to determine the survival of BMSCs in the brain. 2. Due to the difficulty of culture and acquisition of TX mice, the number of animals entered the experiment was small, and only 3 TX mice were included in each observation point. 3. This study did not conduct behavioral comparisons which limited the interpretation of whether histological repair translated into meaningful neurological improvement. The reason is that TX mice have no neurological symptoms but only pathological changes in brain tissue. Subsequent behavioral research may need to be conducted in other animal models. 4. Extrapyramidal neural network reconstruction was carried out using FACT method. This method is an indirect evaluation of neural network. More accurate methods for neural network evaluation should be further sought. 5. The use of mouse derived BMSCs is due to cost concerns as well as ethical reasons. However, the use of mouse derived stem cells cannot fully simulate the function of human stem cells, which will affect the applicability of stem cells in WD patients. Future studies will investigate the role of human stem cells.

Conclusion: BMSCs can ameliorate WD extrapyramidal neural network injury. The recovery effect may be related to BMSCs reducing copper deposition and alleviating oxidative stress and inflammatory response. Behavioral research should be further carried out to confirm whether the repair of neural damage can lead to improvement in symptoms. The mechanism of BMSCs affecting copper metabolism and oxidative stress is still under further study. BMSCs of human origin should be evaluated in the next step and may be considered as regenerative medicine for WD.

## Data Availability

The data analyzed in this study is subject to the following licenses/restrictions: all data generated or analysed during this study are included in this article. Further enquiries can be directed to the corresponding author. Requests to access these datasets should be directed to zxx7317@163.com.
